# The effect of insulin resistance in the association between obesity and hypertension incidence among Chinese middle-aged and older adults: data from China health and retirement longitudinal study (CHARLS)

**DOI:** 10.3389/fpubh.2024.1320918

**Published:** 2024-02-13

**Authors:** Ze-Jiaxin Niu, Ying Cui, Tian Wei, Meng Dou, Bing-Xuan Zheng, Ge Deng, Pu-Xun Tian, Yang Wang

**Affiliations:** ^1^Department of Cardiovascular Medicine, First Affiliated Hospital of Xi’an Jiaotong University, Xi'an, China; ^2^Department of Kidney Transplantation, Hospital of Nephropathy, The First Affiliated Hospital of Xi’an Jiaotong University, Xi'an, China; ^3^Department of Neurological Rehabilitation, North Hospital, Xi’an International Medical Center Hospital, Xi'an, China; ^4^Key Laboratory of Molecular Cardiology of Shaanxi Province, Xi'an, China

**Keywords:** hypertension, obesity, insulin resistance, mediation effects, China health and retirement longitudinal study

## Abstract

**Background and aims:**

Obesity and insulin resistance are well-known important risk factors for hypertension. This study aimed to investigate the mediating effect of the triglyceride-glucose index (TyG) in the association between Chinese visceral obesity index (CVAI) and hypertension among Chinese middle-aged and older adults.

**Methods:**

A total of 10,322 participants aged 45 years and older from CHARLS (2011–2018) were included. Baseline data were collected in 2011 and hypertension incidence data were gathered during follow-up in 2013, 2015 and 2018. Multivariate logistic regression models were constructed to investigate the association of CVAI and TyG with the incidence of hypertension. Additionally, mediation analyses were conducted to evaluate the mediating role of the TyG index in the relationship between CVAI and hypertension. Subgroup analysis was also performed.

**Results:**

A total of 2,802 participants developed hypertension during the follow-up period. CVAI and TyG index were independently and significantly associated with hypertension incidence. Increasing quartiles of CVAI and TyG index were associated with high hypertension incidence in middle-aged and older adults. The TyG index was identified as a mediator in the relationship between CVAI and hypertension incidence, with a mediation effect (95% confidence interval) was 12.38% (6.75, 31.81%).

**Conclusion:**

Our study found that CVAI and TyG were independently associated with hypertension incidence. TyG played a partial mediating effect in the positive association between CVAI and hypertension incidence.

## 1 Introduction

Hypertension, a prominent risk factor for cardiovascular disease, remains a significant public health burden worldwide across various countries and regions (1, 2). Projections indicate that the global incidence of hypertension will reach 29% by 2025, affecting an estimated 1.56 billion individuals (3). Urgent emphasis on preventive measures is required to decrease the incidence of hypertension and the associated healthcare burden. Early and precise identification of risk factors for hypertension, along with the implementation of effective preventive measures, can significantly alleviate the economic strain on healthcare systems (4).

In recent years, there has been increasing evidence that visceral adipose tissue is more closely associated with the onset of hypertension than subcutaneous fat or total fat. Gold standard techniques employed to assess visceral obesity include magnetic resonance imaging (MRI) and computed tomography (CT), however, these methods are not commonly utilized in clinical practice and epidemiological research owing to their inconvenience, high cost and radiation exposure to humans (5). Xia et al. devised the Chinese visceral adiposity index (CVAI) by drawing upon the visceral obesity index (VAI). This index accurately predicts visceral fat content in Chinese populations, aligning with imaging methods (6). Cross-sectional studies carried out among various populations have exhibited a positive association between CVAI and hypertension incidence (7).

Previous studies suggested abnormal glucose-lipid metabolism was significantly associated with the incidence of hypertension, and insulin resistance (IR) also carried a great significance in the pathogenesis of hypertension (8). The gold standard method to assess IR is the clamp/insulin suppression test, but it is too complex to be widely available in clinical settings. Also, this method presents additional challenges due to its cost and limited clinical availability (9). Currently, the triglyceride glucose (TyG) index has been proposed to be a surrogate of insulin resistance due to its easy availability and has been demonstrated as an insulin resistance marker (10, 11). The association between the TyG index and the incidence of hypertension has been confirmed in certain cross-sectional population studies (12–14).

However, there are few studies investigating the association between CVAI and TyG index with hypertension in the Chinese middle-aged and older adults population, especially a lack of studies reporting the mediating role of TyG index in the relationship between CVAI and hypertension. Therefore, the present study was designed to investigate the mediating effect of the TyG index in the CVAI - hypertension association. The data were obtained from the large longitudinal population cohort of the China Health and Retirement Longitudinal Survey (CHARLS).

## 2 Materials and methods

### 2.1 Study cohort

CHARLS is a prospective, nationally representative survey designed to investigate a variety of elements in the personal, familial, financial, health, and communal circumstances of community dwellers in China (15). It was established by the National School of Development at Peking University with funding from the US National Institute on Aging (NIA). In 2011, a baseline survey was conducted with a participant population of 17,705, which included a wide range of topics on demographics, family structure, health status, physical function, and biomarkers. Subsequent surveys were conducted at intervals of 2 to 3 years, with subsequent waves conducted in 2013, 2015, and 2018.

**Figure 1:**
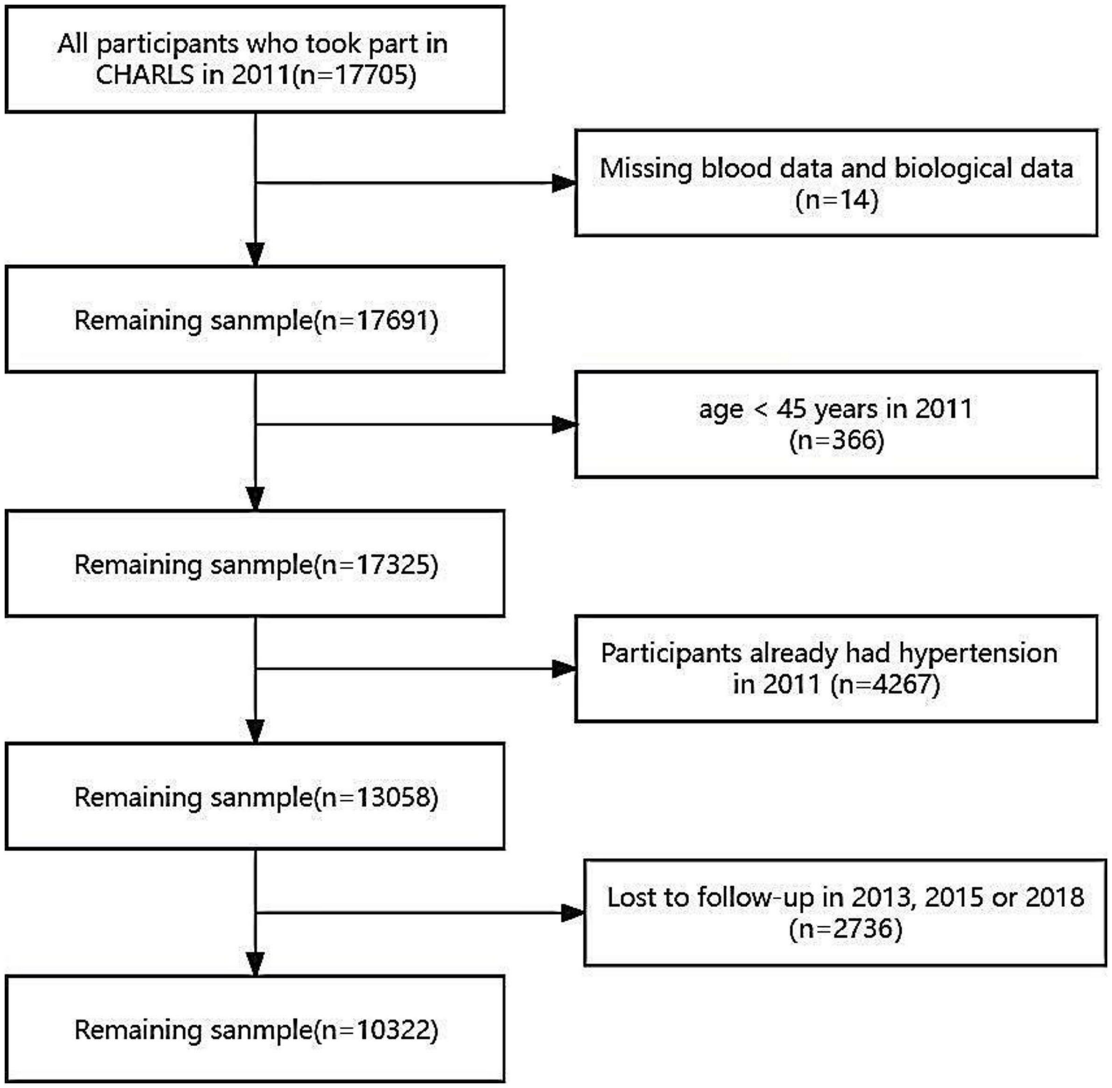
Flow chart for participants’ selection. The 2011 baseline survey accumulated 17,705 respondents, and subjects missing blood samples or biological data (*n* = 14), aged below 45 years (*n* = 366), hypertensive in 2011 (*n* = 4,267) and lost in follow-up (*n* = 2,736) were excluded.

The data for this study were derived from CHARLS baseline dataset collected in 2011, alongside follow-up data on hypertension incidence acquired in 2013, 2015, and 2018. However, participants with the following conditions were excluded: missing blood samples or biological data (*n* = 14), aged below 45 years (*n* = 366), hypertensive in 2011 (*n* = 4,267), or attrition cases (*n* = 2,736; Figure 1). 2,736 participants were lost during the follow-up period, with a loss rate of 15.45%. The loss rate was less than 20%, which was acceptable. The CHARLS research was approved by the Ethics Review Board of Peking University (IRB00001052-11015). Before enrolment, all participants provided written informed consent.

### 2.2 Diagnostic criteria for hypertension, diabetes, dyslipidemia and kidney disease

Hypertension was clinically diagnosed when systolic blood pressure ≥ 140 mmHg and/or diastolic blood pressure ≥ 90 mmHg or the present use of antihypertensive drugs (16). In this study, the following criteria were used to classify subjects as hypertensive or normotensive at baseline and follow-up (2013, 2015 and 2018), respectively: ① It can be self-reported by the patient in response to the question, “Have you been diagnosed with hypertension?”; ② It might be inferred from recent use of antihypertensive medications as informed by the subject in response to, “Are you currently using any antihypertensive medications to manage your blood pressure?”; ③ It could be confirmed from SBP/DBP readings of 140/90 mmHg or more on two or more occasions.

Diabetes was defined as a self-reported history of diabetes: ① It can be self-reported by the patient in response to the question, “Have you been diagnosed with diabetes?”; ② It might be inferred from recent use of medications as informed by the subject in response to, “Are you currently using any medications to manage your blood glucose?”

Dyslipidemia was defined as a self-reported history of dyslipidemia: ① It can be self-reported by the patient in response to the question, “Have you been diagnosed with dyslipidemia?”; ② It might be inferred from recent use of medications as informed by the subject in response to, “Are you currently using any medications to manage your lipids?”

Kidney disease was defined as a self-reported history of kidney disease: ① It can be self-reported by the patient in response to the question, “Have you been diagnosed with kidney disease?”; ② It might be inferred from recent use of medications as informed by the subject in response to, “Are you currently using any medications to manage your renal function?”

### 2.3 Blood biochemical analysis

After an overnight fasting, blood samples were taken by professionals from the Chinese Center for Disease Control and Prevention and stored at −80°C. Participants’ venous blood samples were analyzed for high-density lipoprotein cholesterol (HDL-C), low-density lipoprotein cholesterol (LDL-C), total cholesterol (TC), triglycerides (TG), cystatin C (CysC), uric acid (UA), blood urea nitrogen (BUN) and serum creatinine (Scr) by staff using standard methods.

### 2.4 Laboratory assessment and anthropometric measurements

Trained interviewers individually administered a standardized questionnaire to gather data on the subjects’ demographics, geographic location, medical history, and socioeconomic status. The questionnaire adhered to established protocols. Blood pressure (BP) measurements were performed by the interviewer using a digital sphygmomanometer—Omron^™^ HEM-7112 Monitor. Subjects were instructed to rest for at least 5 min before the blood pressure readings were taken. Three measurements were recorded at 5-min intervals. Weight and height measurements were obtained while participants wore light clothing and no shoes, using an electronic scale model HN-286 from Omron and a stadiometer from Seca Corporation 213. Specialized personnel collected venous blood samples from each participant after an overnight fast, following standard procedures. Waist circumference (WC) was measured with a tape measure at the midpoint between the upper edge of the ilium and the lower edge of the 12th rib, which is the narrowest part of the waist, in a horizontal circle around the abdomen.

The estimated glomerular filtration rate (eGFR), body mass index (BMI), Visceral Adiposity Index (CVAI), and Triglyceride-Glucose (TyG) index were calculated using the following formulas:

1. eGFR (mL/min per 1.73 m^2^) = 175 × serum creatinine (mg/dL) ^-1.234^ × age (years) ^-0.179^ (× 0.79 for girls/females; based on the Modification of Diet in Renal Disease equation and data from Chinese CKD patients) (17);
2. BMI = body weight (kg) /height^2^ (m^2^);
3. CVAI (for males): CVAI = (− 267.93–16.32 × HDL-C [mmol/L] + 0.03 × BMI [kg/m^2^] + 22.00 × log_10_TG [mmol/L] + 0.68 × Age (years) + 4.00 × WC [cm]) and CVAI (for females): CVAI = (− 187.32–11.66 × HDL-C [mmol/L] + 4.32 × BMI [kg/m^2^] + 39.76 × log_10_TG [mmol/L] + 1.71 × Age (years) + 1.12 × WC [cm]);
4. TyG index = ln (TG [mg/dL] × Blood glucose [mg/dL] /2).

### 2.5 Statistical analyses

Quantitative variables (all quantitative variables did not follow a normal distribution) were expressed as median (interquartile range) and qualitative variables were presented as counts and percentages. The Wilcoxon rank sum test and the Pearson chi-square test were utilized for comparing differences between groups. Missing data on all co-variates were imputed using multiple imputation procedures. The Logistic regression models were constructed to analyze the relationship between CVAI and TyG index and hypertension, and all the co-variables with a *p* value less than 0.05 in the univariate logistic regression analysis were included in the final multivariate regression model (including age, sex, marital status, education level, SBP, current smoking and alcohol consumption). Prior to model inclusion, multicollinearity between covariates were assessed using tolerance and the variance inflation factor (VIF). Participants were categorized into four groups (Quartile 1, Quartile 2, Quartile 3, and Quartile 4) based on the quartiles of CVAI and TyG, with Quartile 1 as the reference group. *p* values for trend among quartile groups were determined using the Pearson chi-square test. Two logistic regression models were constructed to analyze the association between the quartile groups of the two indices and the incidence of hypertension. In Model 1, age and sex were included as adjustment covariates following the initial unadjusted analysis. Model 2 further adjusted for marital status, education level, SBP, current smoking, and alcohol consumption after checking for multicollinearity.

Additionally, the general mediation model was constructed to explore the mediating role of the TyG index in the association between CVAI and hypertension. The mediation model adjusted for co-variates, including gender, age, education level, marital status, smoking status, and alcohol consumption. To evaluate the mediation effect, we employed four formulas: (1) C represented the total effect, indicating that CVAI determines hypertension incidence (Y=CX); (2) β1 denoted the indirect effect, illustrating the influence of CVAI on TyG index (M = β1X); (3) c’ signified the direct effect, and β2 denoted the indirect effect, demonstrating how CVAI determined hypertension incidence while controlling for TyG index (Y = β2M + c’X); (4) the indirect effect (βInd) was calculated as β1 × β2. The mediation effect (%) was calculated as βInd/C × 100%. Bootstrapping method (5,000 iterations) was performed to calculate percentile bootstrap 95% confidence intervals (CIs) for direct effect (c’), indirect effect (βInd) and total effect (C = c’ + βInd). In subgroup analysis, participants were stratified according to gender (male or female) age(<65 years old or ≥ 65 years old), smoking status (smokers or non-smokers), BMI (BMI < 24 kg/m^2^, 24 kg/m^2^ ≤ BMI < 28 kg/m^2^, BMI ≥ 28 kg/m^2^), history of diabetes, history of dyslipidemia and history of kidney disease (based on self-reports of participants in 2011) to further evaluate the robustness of the associations between CVAI and the TyG index with the incidence of hypertension. The regression models were performed using the “autoReg” package in R software (version 4.2.1) and the general mediation model was constructed using SPSS software (version 26.0). Statistical significance was set at a two-tailed *p* value of less than 0.05.

## 3 Results

### 3.1 Baseline characteristics of participants

The baseline characteristics and demographic data were shown in Table 1. A total of 10,322 subjects (without hypertension at baseline) were included in the study, of whom 2,802 developed hypertension during the follow-up period. The mean age of the entire subject group was 55 years and 50.1% were male. Compared with those who did not develop hypertension during the follow-up period, those who developed hypertension exhibited significantly higher levels of CVAI, TyG index, SBP, DBP, pulse, WC, blood glucose, HbA1c, BMI, TG, TC, LDL-C, UA, and CysC (all *p* < 0.001). In contrast, participants who developed hypertension had significantly lower HDL-C and eGFR values (*p* < 0.023 and *p* < 0.001; Table 1).

**Table 1:** Baseline characteristics of the participants.

Characteristics	Normotension (*N* = 7,520)	Hypertension (*N* = 2,802)	P*
Age, year	55 (49, 62)	58 (52, 65)	<0.001
Gender (male, %)	3,726 (49.5)	1,450 (51.7)	0.047
Education level (n, %)	–	–	
Below primary school	3,098 (41.3)	1,302 (46.5)	<0.001
Primary or Middle school	3,276 (43.7)	1,196 (42.7)	
High school or above	1,129 (15.0)	301 (10.8)	
Marital status (married, %)	6,792 (90.3)	2,486 (88.7)	0.015
Current smoking (n, %)	3,030 (40.4)	1,193 (42.7)	0.037
Alcohol consumption (n, %)	2,583 (34.4)	1,033 (37.0)	0.016
BMI, kg/m^2^	22.3 (20.3, 24.6)	23.0 (20.7, 25.4)	<0.001
SBP, mmHg	116 (108, 124)	124 (116, 131)	<0.001
DBP, mmHg	70 (64, 76)	73 (67, 79)	<0.001
Pulse, beats/min	71 (65, 78)	72 (66, 79)	<0.001
Waist, cm	82 (76, 88)	84 (78, 91)	<0.001
Blood glucose, mmol/L	100 (93, 110)	103 (95, 114)	<0.001
HbA1c, %	5.19 (4.87, 5.40)	5.29 (4.90, 5.40)	<0.001
TC, mg/dL	186 (164, 210)	192 (166, 217)	<0.001
TG, mg/dL	97 (72, 142)	104 (75, 156)	<0.001
HDL-C, mg/dL	51 (41, 61)	49 (40, 60)	0.023
LDL-C, mg/dL	112 (91, 134)	115 (93, 138)	<0.001
Serum creatinine, mg/dL	0.75 (0.64, 0.87)	0.76 (0.66, 0.88)	0.007
BUN, mg/dL	15.1 (12.5, 18.2)	15.2 (12.5, 18.5)	0.258
eGFR, mL/min/1.73m^2^	98 (88, 105)	96 (86, 104)	<0.001
UA, mg/dL	4.16 (3.47, 4.96)	4.33 (3.60, 5.20)	<0.001
CysC, mg/L	0.95 (0.84, 1.09)	0.97 (0.86, 1.11)	0.005
CVAI	81.27 (57.99, 106.67)	93.40 (68.87, 120.19)	<0.001
TyG	8.50 (8.15, 8.92)	8.60 (8.25, 9.08)	<0.001

*Wilcoxon rank sum test or Pearson's Chi-squared test. Non-normally distributed variables are expressed as the median (inter-quartile range); All other values are expressed as n, %. SBP, systolic blood pressure; DBP, diastolic blood pressure; BMI: body mass index; HbA1c, glycated hemoglobin; BUN, blood urea nitrogen; TC, total cholesterol; TG, triglyceride; HDL-C, high-density lipoprotein cholesterol; LDL-C, low-density lipoprotein cholesterol; UA, uric acid; eGFR, estimated glomerular filtration rate; CysC, cystatin C; CVAI, Chinese visceral adiposity index; TyG, triglyceride glucose index.

### 3.2 Association between CVAI, TyG index and hypertension incidence

CVAI and TyG were significantly associated with hypertension incidence individually by multivariable logistic regression, with odds ratios (OR) and 95% confidence interval (CI) listed for CVAI [1.006 (1.004–1.007), *p* < 0.001] and TyG index [1.15 (1.04–1.28), *p* < 0.001]. The results of the collinearity test in multivariable logistic regressions were presented in Supplementary Table S1.

Table 2 detailed the associations between CVAI and TyG with hypertension incidence by quartile grouping. The quartile groups for CVAI were Q1(<60.98), Q2(60.98–84.72), Q3(84.72–110.84), Q4(>110.84), and the quartile groups for the TyG index were Q1(<8.16), Q2(8.16–8.52), Q3(8.52–9.42), Q4(>9.42). A notable increasing trend in hypertension incidence was observed with the ascending quartile groups of CVAI and TyG (*p* for trend <0.001). Hypertension incidence were lowest in Q1 and highest in Q4 in both the CVAI and TyG quartile groups. In the age-and sex-adjusted models, the ORs and 95% CIs for the CVAI quartile groups were 1.25 (1.04–1.50), 1.43 (1.18–1.72), and 1.91 (1.57–2.32) for the Q2, Q3, and Q4 groups, respectively, compared with the Q1 group (*p* < 0.001). Similarly, for TyG, the ORs (95% CIs) were 1.24 (1.04–1.49), 1.19 (1.01–1.52), and 1.60 (1.26–2.04) for the Q2, Q3, and Q4 groups, respectively (*p* < 0.001). In the multivariate model, the incidence of hypertension increased with CVAI quartiles, with the ORs (95% CIs) being 1.26 (1.05–1.51), 1.45 (1.20–1.75), and 1.93 (1.59–2.35; *p* < 0.001), respectively. Similarly, the incidence of hypertension increased by 1.25 (1.04–1.49), 1.19 (1.01–1.42), and 1.63 (1.28–2.07; *p* < 0.001), respectively, with increasing quartiles of the TyG index. The goodness-of-fit of the logistic regression models was assessed using the Akaike information criterion (AIC), with the AIC of the multiple-adjusted logistic regression model being significantly lower than that of the age-sex-adjusted regression model, suggesting that the multiple-adjusted regression model offered a better fit.

**Table 2:** Association between quartiles of CVAI, TyG and the incidence of hypertension.

	Case (%)	Age, sex-adjusted			Multivariate*		
		OR (95%CI)	p	AIC	OR (95%CI)	p	AIC
CVAI							
Q1(<60.98)	281(21.1%)	1.00(reference)	–	6,233	1.00(reference)	–	5,811
Q2(60.98–84.72)	341 (25.5%)	1.25 (1.04–1.50)	0.019		1.26 (1.05–1.51)	0.015	
Q3(84.72–110.84)	391 (29.5%)	1.43 (1.18–1.72)	<0.001		1.45 (1.20–1.75)	<0.001	
Q4(>110.84)	510 (38.1%)	1.91 (1.57–2.32)	<0.001		1.93 (1.59–2.35)	<0.001	
*P* for trend	<0.001	–	–		–	–	–
TyG							
Q1(<8.16)	383 (23.9%)	1.00(reference)	–	7,793	1.00(reference)	–	5,901
Q2(8.16–8.52)	474 (28.8%)	1.24 (1.04–1.49)	0.017		1.25 (1.04–1.49)	0.016	
Q3(8.52–9.42)	796 (30.1%)	1.19 (1.01–1.52)	0.043		1.19 (1.01–1.42)	0.040	
Q4(>9.42)	258 (37.3%)	1.60 (1.26–2.04)	<0.001		1.63 (1.28–2.07)	<0.001	
*P* for trend	<0.001	–	–		–	–	

*Logistic regression analyses were used after adjustment for age, sex, marital status, education level, SBP, current smoking and alcohol consumption. *p* values for trend among quartile groups were determined using Pearson chi-square test. To avoid covariance in multivariate logistic regression analysis, waist circumference, blood glucose, triglycerides, total cholesterol and LDL-C were excluded. OR, odds ratio; CI, confidence interval; AIC, Akaike information criterion; CVAI, Chinese visceral adiposity index; TyG, triglyceride glucose index.

### 3.3 The mediating effect of TyG on the association between CVAI and hypertension incidence

**Figure 2:**
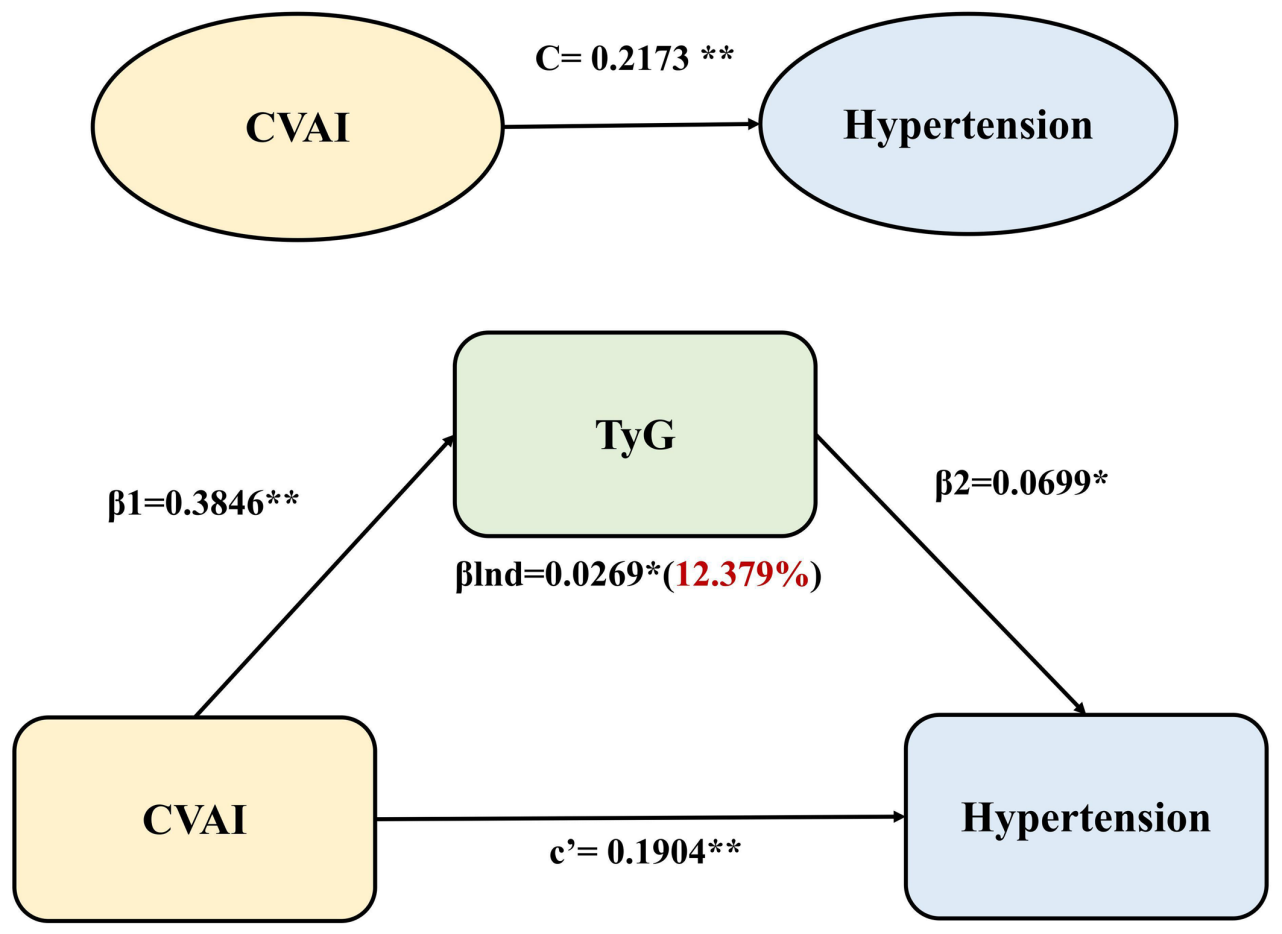
Mediation effect of TyG in the associations between CVAI and hypertension incidence using the general mediation model. The regression coefficient β1 represented the effect of CVAI on TyG index, the regression coefficient β2 represented the effect of TyG on hypertension incidence, βlnd (indirect effect) was calculated as β1 × β2, c’ signified the direct effect of CVAI on hypertension, and C represented the total effect of CVAI on hypertension (the sum of the direct and indirect effects). **p < 0.001,*p < 0.05.

After adjusting age, sex, marital status, education level, current smoking and alcohol consumption, the TyG index partially mediated the effect of CVAI on hypertension in the general mediation model. The regression coefficient β1 representing the effect of CVAI on the TyG index was 0.3846 (*p* < 0.001), and the regression coefficient β2 signifying the effect of TyG on hypertension incidence was 0.0699 (*p* < 0.05). βlnd representing the effect of CVAI on the incidence of hypertension mediated by TyG (indirect effect) was 0.0269 (*p* < 0.05). Significant direct effect (c’), indirect effect (βInd) and total effect (C) were observed in mediation analyses (all *p* < 0.05; Figure 2). The coefficients and 95%CIs of total effect, direct effect and indirect effect were 0.2173 (0.1555, 0.2770), 0.1904 (0.1450, 0.2359) and 0.0269 (0.0105, 0.0411), respectively. The TyG index emerged as the mediator in the association between CVAI and hypertension incidence and the percentage of the mediating effect of the TyG index was 12.38% (95%CI: 6.75 to 31.81%; Supplementary Table S2).

### 3.4 Subgroup analysis

**Figure 3:**
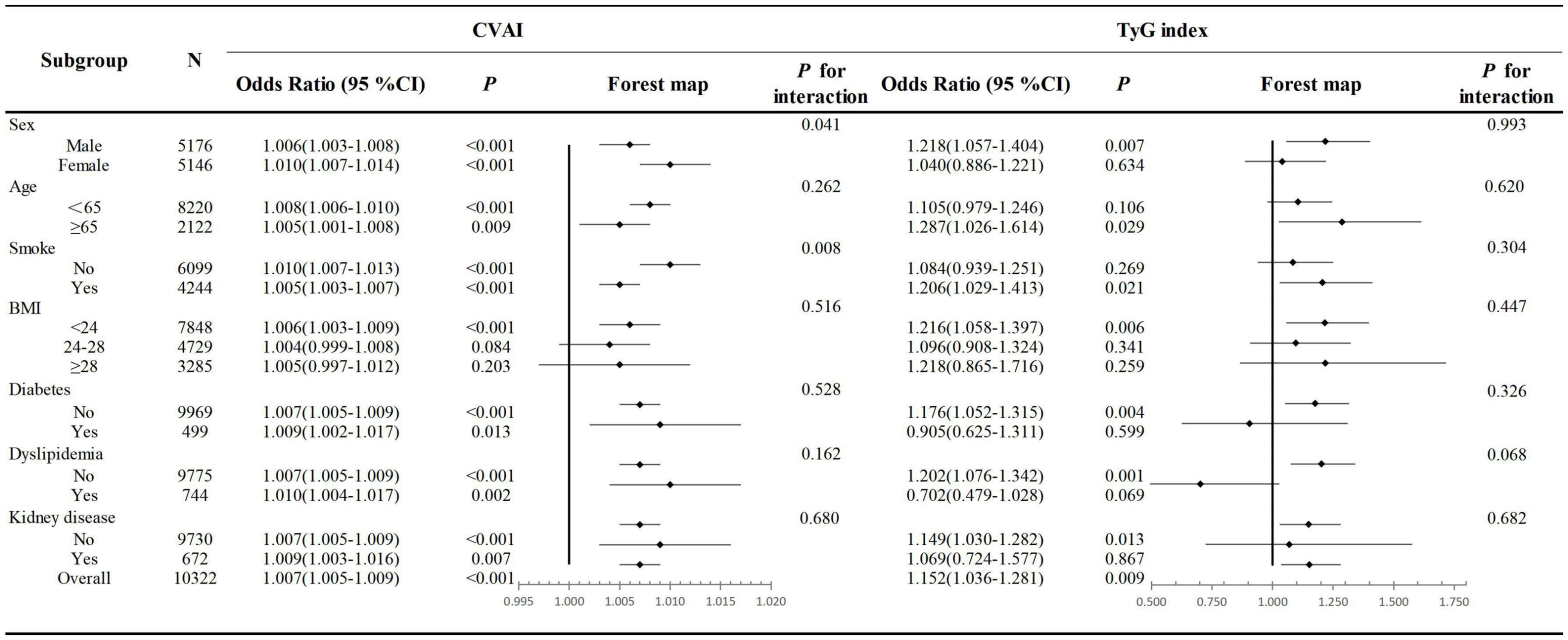
Association of the CVAI and TyG index and hypertension incidence in different subgroups. Multivariate logistic regression models were adjusted for age, sex, marital status, education level, SBP current smoking and alcohol consumption. OR, odds ratio; CI, confidence interval; CVAI, Chinese visceral adiposity index; TyG, triglyceride glucose index.

To confirm the robustness of our findings, we conducted several subgroup analyses. The adjusted ORs and 95% CIs for hypertension incidence associated with CVAI and TyG index across various subgroups were illustrated in Figure 3. Significant associations between CVAI and hypertension were observed in all subgroups except those with BMI ≥ 24 kg/m^2^. TyG index was significantly associated with hypertension in participants aged >65 years, males, smokers, those with a BMI < 24 kg/m^2^, subgroups without diabetes, dyslipidemia, and kidney disease(all *p* < 0.05). Additionally, CVAI had no interaction with age, sex, BMI, diabetes history, dyslipidemia and chronic kidney disease (all *p* for interaction >0.05). There also was no interaction observed between the TyG index and any of the subgroup variables (all *p* for interaction >0.05).

## 4 Discussion

This study explored the complex inter-associations between obesity, insulin resistance, and hypertension among Chinese middle-aged and older adults from a large Chinese population-based cohort. Our study highlights several important findings: the higher the level of CVAI or TyG index, the higher the incidence of hypertension. Both CVAI and TyG index were significantly associated with the incidence of hypertension. More importantly, the TyG index was identified as a mediator in the relationship between CVAI and hypertension incidence. Thus, these findings provided evidence for elucidating the relationship between obesity, insulin resistance and hypertension based on the population-based cohort data.

Dyslipidemia and overweight or obesity are widely recognized as the most prevalent risk factors for hypertension (18–22). Recent studies have shown that visceral adipose tissue (VAT) is more strongly associated with the development of hypertension than subcutaneous abdominal adipose tissue (23, 24), The CVAI index has emerged as a straightforward and reliable indicator of visceral adiposity in Chinese individuals and is gaining attention due to its association with hypertension. In comparison with waist-to-hip ratio, BMI, and lipid accumulation product (LAP), the CVAI has demonstrated greater accuracy in assessing visceral adiposity in Chinese populations (13, 25, 26). A cross-sectional study involving 9,488 Chinese participants, which compared the predictive value of CVAI with other obesity indices including BMI, WC, VAI, LAP, waist-to-height ratio, body shape index, and body roundness index, revealed that the CVAI was the most reliable predictor of hypertension incidence (25). Additionally, Han and colleagues found that the CVAI exhibited superior predictive performance for hypertension among 9,359 rural residents in the Henan Province of China (27). Furthermore, an analysis of the Framingham Heart Study’s third-generation cohort revealed a positive association between a reduction in VAT attenuation and an increased incidence of hypertension, even after adjusting for BMI and WC (28). Our findings were consistent with previous studies that CVAI was independently associated with hypertension in middle-aged and older adults.

Similarly, insulin resistance (IR) is recognized as a crucial factor in the pathogenesis of hypertension (19, 20, 29), previous studies have consistently demonstrated the high sensitivity of the TyG index in identifying insulin resistance. Additionally, these studies have revealed significant and independent positive associations between TyG indexes and both BP levels and hypertension (30–36), A longitudinal, population-based cohort study spanning 9 years demonstrated significant associations between higher TyG indices and a higher incidence of hypertension in Chinese individuals, albeit being limited to only men (37). Another cross-sectional study based on children and adolescents showed a strong association between elevated TyG index and the incidence of prehypertension and hypertension (38). Further studies have elucidated that TyG index is closely related to target organ damage and adverse cardiovascular events in patients with essential hypertension (39, 40). Our study also demonstrated a significant increase in the incidence of hypertension with an increase in the TyG index in middle-aged and older adults. However, the association between TyG and hypertension was inconsistent in subgroup analyses. Insulin sensitivity is influenced by various factors, including age (41, 42), gender (43), smoking (44, 45), abnormal glucose and lipid metabolism (46, 47) and chronic kidney diseases (48). As an important marker of insulin resistance, the TyG index is likely affected by these factors, resulting in inconsistent associations between the TyG index and hypertension in subgroup analyses.

Obesity and insulin resistance, both recognized as major risk factors for the development of hypertension, exhibit a robust association with each other. Previous studies have reported on the complex inter-relationships between obesity, insulin resistance, and the incidence of hypertension (19, 20, 29). Visceral fat, characterized by a higher prevalence of large adipocytes compared to subcutaneous fat, contributes to adipocyte dysfunction during their enlargement. These large adipocytes are highly insulin-resistant, resulting in endothelial (49) and continuous activation of the angiotensin-aldosterone system (49), sodium retention (50), increased sympathetic nerve activity (51), and atherosclerotic stenosis, all of which are significant contributors to the development of hypertension (52–55). Thus, obesity may contribute to hypertension by inducing insulin resistance. Our study expanded previous findings by demonstrating the partial mediation effects of the TyG index in the association between CVAI and the incidence of hypertension in middle-aged and older adults, with a mediation effect of 12.38%. This insight provided evidence that obesity and hypertension are linked through insulin resistance.

The main strength of this study lies in its exploration of the TyG index as a mediator between CVAI and hypertension incidence, which provides a basis for elucidating the complex relationship between obesity, insulin resistance and hypertension. However, our study has certain limitations. Firstly, although adjustment for various covariates, we were unable to adjust for other confounders such as environmental and psychological factors. Secondly, imaging data, which would allow for a more precise evaluation of visceral fat distribution, were not collected from participants in the CHARLS population cohort. Lastly, we used CVAI as an assessment of visceral fat distribution in Chinese, which somewhat limits the applicability of the results of this study to other Asian populations.

In conclusion, our findings demonstrated that CVAI and TyG index were independently and significantly associated with hypertension incidence among Chinese middle-aged and older adults data from CHARLS. More importantly, our study also revealed that TyG index acted as a partial mediating role in the association between CVAI and hypertension incidence. This insights contributed significantly to understanding of the role of insulin resistance in the relationship between obesity and hypertension.

## Data availability statement

The original contributions presented in the study are included in the article/Supplementary material, further inquiries can be directed to the corresponding authors.

## Ethics statement

The studies involving humans were approved by the Biomedical Ethics Review Committee of Peking University (IRB00001052-11015). The studies were conducted in accordance with the local legislation and institutional requirements. The participants provided their written informed consent to participate in this study. Written informed consent was obtained from the individual(s) for the publication of any potentially identifiable images or data included in this article.

## Author contributions

Z-JN: Data curation, Formal analysis, Investigation, Methodology, Resources, Writing – original draft, Writing – review & editing. YC: Data curation, Resources, Software, Writing – original draft. TW: Investigation, Writing – original draft. MD: Formal analysis, Investigation, Writing – original draft. B-XZ: Data curation, Formal analysis, Investigation, Writing – review & editing. GD: Writing – review & editing, Data curation, Formal analysis, Investigation. P-XT: Writing – review & editing, Writing – original draft. YW: Writing – original draft, Writing – review & editing, Methodology, Supervision.
