# Supplementary material for: The effect of insulin resistance in the association between obesity and hypertension incidence among Chinese middle-aged and older adults: data from China health and retirement longitudinal study (CHARLS)
Source: Front Public Health. 2024 Feb 13;12:1320918. doi: 10.3389/fpubh.2024.1320918 (PMC10898648; doi:10.3389/fpubh.2024.1320918)
Supplement: Supplementary file 1 [file Table_1.docx]

Supplementary Material

# Supplementary Table

# Table S1. Collinearity test in multifactor logistic regressions

| **Multiple factors** | **CVAI** | | **TyG** | |
| --- | --- | --- | --- | --- |
|  | **VIF** | **Tolerance** | **VIF** | **Tolerance** |
| Age | 1.106271 | 0.9039380 | 1.100207 | 0.9089202 |
| Education | 1.045959 | 0.9560603 | 1.045446 | 0.9565292 |
| Marital | 1.046135 | 0.9559000 | 1.046488 | 0.9555773 |
| Gender | 1.508381 | 0.6629625 | 1.501781 | 0.6658762 |
| SBP | 1.014659 | 0.9855529 | 1.011487 | 0.9886438 |
| Smoke | 1.392962 | 0.7178947 | 1.388187 | 0.7203641 |
| Drink | 1.146818 | 0.8719780 | 1.146225 | 0.8724290 |
| CVAI | 1.024965 | 0.9756428 | - | - |
| TyG | - | - | 1.009538 | 0.9905518 |

The variance inflation factor test was used to evaluate the collinearity problem respectively. VIF, variance inflation factor. VIF of all parameters was less than 10, indicating that there was no serious collinearity problem.

Table S2 Mediation analyses of TyG index in the associations between CVAI and hypertension

| **Independent variable** | **Mediator** | **Total effect** | | **Indirect effect** | | **Direct effect** | | **Proportion mediated, % (95% CI)** |
| --- | --- | --- | --- | --- | --- | --- | --- | --- |
|  |  | **Coefficient**  **(95% CI)** | ***P*** | **Coefficient**  **(95% CI)** | ***P*** | **Coefficient**  **(95% CI)** | ***P*** |  |
| CVAI | TyG | 0.2173  (0.1555, 0.2770) | <0.001 | 0.0269  (0.0105, 0.0411) | 0.0105 | 0.1904  (0.1450, 0.2359) | <0.001 | 12.38  (6.75, 31.81) |

The mediation analyses were adjusted for age, sex, marital status, education level, current smoking and alcohol consumption. CI, confidence interval; CVAI, Chinese visceral adiposity index; TyG, triglyceride glucose index; The effect of CVAI on the incidence of hypertension is the direct effect, the effect of CVAI on the incidence of hypertension via TyG is the indirect effect, and the sum of the two is the total effect.

**Table S3. STROBE guideline (2019) based on this study**

|  | **Recommendation** | **Pagination** |
| --- | --- | --- |
| **Title and abstract** | 1. Indicate the study's design with a commonly used term in the title or the abstract 2. Provide in the abstract an informative and balanced summary of what was done and what was found | Line 24-42 |
| **Introduction** |  |  |
| Background/rationale | Explain the scientific background and rationale for the investigation being reported | Line 46-68 |
| Objectives | State specific objectives, including any pre-specified hypotheses | Line 69-74 |
| **Methods** |  |  |
| Study design | Present key elements of study design early in the paper | Line 79-84 |
| Setting | Describe the setting, locations, and relevant dates, including periods of recruitment, exposure, follow-up, and data collection | Line 85-90 |
| Participants | 1. Cohort study—Give the eligibility criteria, and the sources and methods of selection of participants. Describe methods of follow-up   Case-control study-Give the eligibility criteria, and the sources and methods of case ascertainment and control selection. Give the rationale for the choice of cases and controls  Cross-sectional study-Give the eligibility criteria, and the sources methods of selection of participants | Line 86-88  Figure 1 |
|  | 1. Cohort study-For matched studies, give matching criteria and number of exposed and unexposed   Case-control study-For matched studies, give matching criteria and the number of controls per case | - |
| Variables | Clearly define all outcomes, exposures, predictors, potential confounders, and effect modifiers. Give diagnostic criteria, if applicable | Line 94-143 |
| Data sources/ measurement | For each variable of interest, give sources of data and details of methods of assessment (measurement). Describe comparability of assessment methods if there is more than one group | Line 121-143 |
| Bias | Describe any efforts to address potential sources of bias | - |
| Study size | Explain how the study size was arrived at | - |
| Quantitative variables | Explain how quantitative variables were handled in the analyses. If applicable, describe which groupings were chosen and why | Line 145-146 |
| Statistical methods | 1. Describe all statistical methods, including those used to control for confounding | Line 149-161 |
|  | 1. Describe any methods used to examine subgroups and interactions | Line 172-180 |
|  | 1. Explain how missing data were addressed | Line 148-149 |
|  | 1. Cohort study-If applicable, explain how loss to follow-up was addressed   Case-control study-If applicable, explain how matching of cases and controls was addressed | Line 138-140 |
|  | 1. Describe any sensitivity analyses | Line 172-180 |
| **Results** |  |  |
| Participants | 1. Report numbers of individuals at each stage of study— e.g. numbers potentially eligible, examined for eligibility, confirmed eligible, included in the study, completing follow-up, and analyzed 2. Give reasons for non-participation at each stage 3. Consider use of a flow diagram | Line 86-88  Figure 1 |
| Descriptive data | 1. Give characteristics of study participants (e.g. demographic, clinical, social) and information on exposures and potential confounders 2. Indicate number of participants with missing data for each variable of interest 3. Cohort study—Summarize follow-up time (e.g. average and total amount) | Line 184-190  Table 1 |
| Outcome data | Cohort study—Report numbers of outcome events or summary measures over time | Line 183-185 |
|  | Case-control study—Report numbers in each exposure category, or summary measures of exposure |  |
|  | Cross-sectional study-Report numbers of outcome events or summary measures |  |
| Main results | 1. Give unadjusted estimates and, if applicable, confounder-adjusted estimates and their precision (e.g., 95% confidence interval). Make clear which confounders were adjusted for and why they were included 2. Report category boundaries when continuous variables were categorized 3. If relevant, consider translating estimates of relative risk into absolute risk for a meaningful time period | Line 196-225 |
| Other analyses | Report other analyses done—e.g. analyses of subgroups and interactions, and sensitivity analyses | Line 227-235 |
| **Discussion** |  |  |
| Key results | Summarize key results with reference to study objectives | Line 239-244 |
| Limitations | Discuss limitations of the study, taking into account sources of potential bias or imprecision | Line 293-298 |
|  | Discuss both direction and magnitude of any potential bias |  |
| Interpretation | Give a cautious overall interpretation of results considering objectives, limitations, multiplicity of analyses, results from similar studies, and other relevant evidence | Line 245-290 |
| Generalisability | Discuss the generalisability (external validity) of the study results | Line 297-298 |
| **Other information** |  |  |
| Funding | Give the source of funding and the role of the funders for the present study and, if applicable, for the original study on which the present article is based | Lin319-325 |
